# Tissue S100/calgranulin expression and blood neutrophil-to-lymphocyte ratio (NLR) in prostatic disorders in dogs

**DOI:** 10.1186/s12917-023-03792-0

**Published:** 2023-11-09

**Authors:** Jana Weinekötter, Corinne Gurtner, Martina Protschka, Wolf von Bomhard, Denny Böttcher, Gottfried Alber, Ingmar Kiefer, Joerg M. Steiner, Johannes Seeger, Romy M. Heilmann

**Affiliations:** 1 Department for Small Animals, College of Veterinary Medicine, Leipzig University, An den Tierkliniken 23, DE-04103 Leipzig, SN Germany; 2 Institute of Animal Pathology, Department of Infectious Diseases and Pathobiology, Vetsuisse Faculty, University of Bern, Länggassstrasse 122, Bern, BE CH-3001 Switzerland; 3 Institute of Immunology, College of Veterinary Medicine, Biotechnological-Biomedical Center, Leipzig University, Deutscher Platz 05, DE-04103 Leipzig, SN Germany; 4 Synlab Specialty Center for Veterinary Pathology, Hartelstrasse 30, DE-80689 Munich, BY Germany; 5 Institut for Veterinary Pathology, College of Veterinary Medicine, Leipzig University, An den Tierkliniken 33, DE-04103 Leipzig, SN Germany; 6 Gastrointestinal Laboratory, Department of Small Animal Clinical Sciences, School of Veterinary Medicine and Biomedical Sciences, Texas A&M University, TAMU, College Station, TX 4474, 77843-4474 USA; 7 Institute of Anatomy, Histology and Embryology, College of Veterinary Medicine, Leipzig University, An den Tierkliniken 43, DE-04103 Leipzig, SN Germany

**Keywords:** Biomarker, Calprotectin, Diagnostic accuracy, S100A8/A9, S100A12, Prostatic carcinoma, Benign prostate hyperplasia, Prostatitis

## Abstract

**Background:**

Prostatic carcinoma (PCA) is a rare but severe condition in dogs that is similar to the androgen-independent form of PCA in men. In contrast to humans, PCA is difficult to diagnose in dogs as reliable biomarkers, available for PCA screening in human medicine, are currently lacking in small animal oncology. Calprotectin (S100A8/A9) and S100A12 are Ca^2+^-binding proteins of the innate immune system with promising potential to distinguish malignant from benign urogenital tract conditions, similar to the blood neutrophil-to-lymphocyte-ratio (NLR). However, both have not yet been extensively investigated in dogs with PCA. Thus, this study aimed to evaluate the expression of the S100/calgranulins (calprotectin, S100A12, and their ratio [Cal-ratio]) in prostatic biopsies from nine dogs with PCA and compare them to those in dogs with benign prostatic lesions (eight dogs with prostatitis and ten dogs with benign prostatic hyperplasia [BPH]) as well as five healthy controls. In addition, blood NLRs were investigated in twelve dogs with PCA and 22 dogs with benign prostatic conditions.

**Results:**

Tissue S100A8/A9^+^ cell counts did not differ significantly between tissue from PCA and prostatitis cases (*P* = 0.0659) but were significantly higher in dogs with prostatitis than BPH (*P* = 0.0013) or controls (*P* = 0.0033). S100A12^+^ cell counts were significantly lower in PCA tissues than in prostatitis tissue (*P* = 0.0458) but did not differ compared to BPH tissue (*P* = 0.6499) or tissue from controls (*P* = 0.0622). Cal-ratios did not differ significantly among the groups but were highest in prostatitis tissues and significantly higher in those dogs with poor prostatitis outcomes than in patients that were still alive at the end of the study (*P* = 0.0455). Blood NLR strongly correlated with prostatic tissue S100A8/A9^+^ cell counts in dogs with PCA (ρ = 0.81, *P* = 0.0499) but did not differ among the disease groups of dogs.

**Conclusions:**

This study suggests that the S100/calgranulins play a role in malignant (PCA) and benign (prostatic inflammation) prostatic conditions and supports previous results in lower urinary tract conditions in dogs. These molecules might be linked to the inflammatory environment with potential effects on the inflammasome. The blood NLR does not appear to aid in distinguishing prostatic conditions in dogs. Further investigation of the S100/calgranulin pathways and their role in modulation of tumor development, progression, and metastasis in PCA is warranted.

## Background

Prostatic carcinoma (PCA) is the most common non-skin-associated neoplasia and the second most common reason for cancer-associated death in the United States in men [1]. While arising from the epithelium and mostly growing as adenocarcinoma, ascending urothelial carcinoma (UC) can also occur. Dogs with PCA have a great potential to serve as models for human PCA because they are, in addition to other non-human primates, the only larger mammal bearing naturally occurring PCA [2, 3]. Men and dogs share similarities in the anatomy and function of the prostate [4], but some differences exist in the tumor pathobiology between both species. PCA can be (i) androgen-dependent, as is mostly the case in men [5], or (ii) androgen-independent, being the most common form in dogs and carrying a very poor prognosis compared to androgen-dependent PCA, which has a more favorable disease course in most cases. Hence, neutered dogs with PCA are the most relevant model for advanced androgen-independent PCA in men [6].

In dogs, the age of PCA onset is similar to those affected by UC [7], and neutering appears to be one of the main risk factors for developing PCA [8–12]. In affected intact male dogs, however, PCA is often even more aggressive, with Doberman pinschers and Airedale terriers showing an increased risk for developing PCA compared to other breeds [7, 12]. More than 40% of canine PCA patients have metastatic disease at diagnosis, and this proportion increases to 80% at the time of death. Typical locations for metastases are the regional lymph nodes, spleen, liver, and the lungs, but bone metastasis to the limbs, pelvis, and lumbar spine can also occur and result in lameness or neurologic signs [4, 9, 13–15]. Clinical signs of PCA are not specific and include tenesmus, dysuria or stranguria, hematuria, and weight loss, often mimicking UC or urinary tract infection (UTI). Dyschezia is often the main presenting complaint when the prostate is enlarged enough to compromise large intestinal transit [7, 9].

Diagnosis and treatment of PCA can be challenging. The diagnostic gold standard is cytology and/or histopathology, both requiring prostate sampling by biopsy, prostate suction (traumatic catheterization or prostatic wash), or fine-needle aspirate with the dog under general anesthesia or well-sedated [10]. The prostate-specific antigen (PSA) can be measured in the blood to screen for PCA and can also be used in immunohistochemistry (IHC) to help distinguish between prostatic UC and PCA and is thus considered a reliable biomarker in human medicine [16]. However, in dogs, neither canine prostate-specific esterase (CPSE), PSA, nor the acid phosphatase (AP) test can help to distinguish BPH from PCA [17–19]. The detection of *BRAF* polymorphisms showed promising diagnostic value as the specificity in urine samples is 100%, but approximately 20% of PCAs in dogs are not associated with a *BRAF* mutation resulting in a sensitivity of ~ 80% to detect PCA with urine samples. Thus, a reliable screening marker for dogs with PCA is currently lacking [20, 21]. In addition, most medical treatment options are not sufficiently successful, of which non-steroidal anti-inflammatory drugs (NSAIDs) present agents with acceptable side effects [22]. Still, the median survival time does not exceed 6.9 months [22], and novel diagnostic and therapeutic targets are urgently needed.

S100A8/A9 (calprotectin or calgranulin A/B complex) and S100A12 are Ca^2+^-binding proteins of the innate immune response [23]. There is increasing interest in the diagnostic and therapeutic role of these S100/calgranulin proteins and their downstream pathways in dogs [24–27] as well as in human medicine [23, 28–33]. However, their diagnostic use in veterinary medicine is currently limited owing to the lack of assays that are widely available. Previous studies have proposed that the S100/calgranulins and their ratio are promising urinary biomarkers that can distinguish inflammatory from neoplastic conditions of the canine urogenital tract, but expression and localization of these molecules in corresponding tissues have not yet been reported [25].

The neutrophil-to-lymphocyte ratio (NLR) is an easily available marker requiring a complete blood cell count (CBC). The diagnostic and/or prognostic potential of blood NLR has been shown in human patients with inflammatory and neoplastic diseases [34–37]. The blood NLR is also considered a useful biomarker in veterinary medicine and shows promise as an indicator of outcomes in several inflammatory [38–42] and neoplastic conditions [42–45], including chronic inflammatory enteropathy [40], pneumonia [38], acute diarrhea in puppies [41], soft tissue sarcoma [44], and multicentric lymphoma [43]. A recent study compared these findings with the IHC investigation of the S100/calgranulins in the urinary bladder and urethral tissues, showing discrepancies between urinary calgranulin levels and the numbers of S100/calgranulin-expressing cells [42]. In contrast to urinary specimens, no significant difference in the number of S100/calgranulin-positive cells was found between inflammatory and neoplastic diseases, but the blood NLR differentiated both groups [42].

Based on the hypothesis that the S100/calgranulins are also involved in the pathogenesis of PCA, this study aimed to compare S100A8/A9 and S100A12 immunostaining between tissues of prostatic neoplasia, marked prostatitis, benign prostatic hyperplasia (BPH, in some cases accompanied by minimal to mild chronic non-suppurative prostatitis), and a healthy prostate. In dogs with prostatic neoplasia, the possibility of an association between tissue S100/calgranulin expression and indicators of disease severity and outcomes was also tested. Furthermore, the study examined possible differences in the blood NLR between these prostatic disease groups.

## Results

### Study population

*Dogs with prostatic carcinoma* – Breeds included Rhodesian Ridgeback (n = 2), Airedale terrier, Bernese Mountain dog, Fox terrier, Jack Russel terrier, Labrador retriever, Maltese (each n = 1), and mixed breed (n = 6). Most dogs in this group were neutered (n = 10; 71%) (Table 1). PCA (n = 10) or UC (n = 4) was histologically confirmed in all prostatic tissue biopsies obtained via ultrasound-guided TruCut (USGTC) biopsy using a 16G spring-loaded instrument (n = 11) or laparotomy (n = 3). One dog was diagnosed with secondary peritonitis and lymphangitic carcinomatosis, and regional lymph node metastasis was confirmed or suspected in another four dogs. Five dogs had ultrasonographic or radiographic lesions suspicious for distant metastasis to the spleen (n = 3), liver (n = 1), and/or lung (n = 2); however, these lesions were not sampled. Bacterial urine culture was performed in seven dogs, one (14%) being positive for *Streptococcus canis*, and in bacterial cultures of prostatic tissue from eight dogs, one (13%) culture was positive for *Enterobacter cloacae*. Treatment at the time of tissue biopsy included NSAIDs (robenacoxib: n = 2; carprofen: n = 2, cimicoxib: n = 1, meloxicam: n = 1) and/or antimicrobials (amoxicillin: n = 1; amoxicillin/clavulanic acid: n = 1; enrofloxacin: n = 1; sulfadimidine: n = 1; other: n = 3). At the conclusion of the study follow-up time, all dogs in this group had been euthanized (n = 13) or died (n = 1) between 0 and 665 days after diagnosis; only four dogs (29%) lived > 1 month after diagnosis.

**Table 1:** Patient characteristics of all dogs included in the study (n = 45)

Parameter	Prostatic neoplasia	Prostatitis	Benign prostatic hyperplasia	Control dogs	*P* value^†^
Total number of dogs for IHC analyses for NLR analysis	14 9 12	14 8 13	12 10 9	5 5 –	–
Age^†^, in years median [range]	**10.3** ^A^ [7.3–12.8]	**10.0** ^A^ [4.7–13.2]	**9.2** ^A,B^ [1.8–12.0]	**5.0** ^B^ [2.0–10.0]	**0.0431**
Weight^†^, in kg median [range]	**20.9** ^A^ [4.7–45.0]	**23.5** ^A^ [7.0–37.0]	**35.3** ^B^ [19.6–47.0]	**21.8** ^A^ [5.2–27.0]	**0.0258**
Neuter status^†^, n (%) intact / neutered male	4 (29%) / **10 (71%)**^**A**^	10 (71%) / 4 **(29%)**^**A,B**^	10 (83%) / 2 (17%)^B^	5 (100%) / **0**^**B**^	**0.0026**
Breed pure-bred mixed breed	8 (57%) 6 (43%)	9 (64%) 5 (36%)	8 (67%) 4 (33%)	4 (80%) 1 (20%)	0.8200
Sample type USGTC surgical biopsy post-mortem	11 (79%) 3 (21%) 0	7 (50%) 7 (50%) 0	9 (75%) 3 (25%) 0	0 0 5 (100%)	–
NSAID treatment treatment-naïve prior NSAID treatment	8 (57%) 6 (43%)	7 (50%) 7 (50%)	9 (75%) 3 (25%)	–	–
Urine culture^$^ positive negative	1 (14%) 6 (86%)	7 (64%) 4 (36%)	4 (67%) 2 (33%)	–	–
Prostatic tissue culture^#^ positive negative	1 (12%) 7 (88%)	5 (56%) 4 (44%)	0 2 (100%)	–	
Survival time^†^, in days median [range]	**14** ^A^ [0–665]	**682** ^B^ [6–2,146]	**1,741** ^C^ [249–3,230]	–	**< 0.0001**

USGTC = ultrasound-guided TruCut biopsy; ^$^available from 24 dogs; ^#^available from 19 dogs; ^†^bold font indicates significant differences or associations at *P* < 0.05 (values with a different superscript indicate significance at *P* < 0.05)

*Dogs with prostatitis* – Breeds included Beagle (n = 2), German Shorthair pointer, Iceland dog, Lhasa Apso, Malinois, Miniature schnauzer, Rhodesian Ridgeback, Tibet terrier (each n = 1), and mixed breed (n = 5). Four of the dogs were neutered (29%). Histological diagnoses included severe acute suppurative prostatitis (n = 4), chronic suppurative prostatitis with abscessation (n = 1) or cyst formation (n = 8), and paraprostatic cyst with severe inflammation (n = 1). Urine culture, performed in eleven dogs, was positive for bacterial growth in 7/11 (64%), which grew *Escherichia coli* (n = 5), *Enterobacter aerogenes* (n = 1), or *Staphylococcus intermedius* (n = 1). Prostatic bacterial culture was performed in nine dogs, revealing five (56%) positive results (*E. coli*: n = 3, *E. aerogenes*: n = 1, and *Staphylococcus aureus*: n = 1). Five dogs were treatment-naïve while the remaining nine dogs had received NSAID (meloxicam: n = 6; carprofen: n = 1) and/or antimicrobials (amoxicillin/clavulanic acid: n = 4; marbofloxacin: n = 2; metronidazole: n = 1). Seven tissue biopsy samples were surgically obtained, and the remaining seven tissues samples were acquired by USGTC biopsy.

*BPH* – Breeds included English bulldog (n = 2), German Shepherd dog (n = 2), American bulldog, Bernese Mountain dog, Malinois, Weimaraner (each n = 1), and mixed breed (n = 4). Most dogs (n = 10; 83%) were intact. Three prostatic tissue samples were obtained during laparotomy, and nine prostatic specimens via USGTC biopsy. Bacterial urine culture was performed in six dogs, four (67%) being positive (*E. coli*, *Klebsiella ornitholytica*, *S. canis*, or *S. intermedius*), and bacterial culture of prostatic tissue from 2 dogs, both of which were negative for bacterial growth. Two dogs were naïve to any medical treatment, while the remaining dogs had received an NSAID (firocoxib: n = 1; robenacoxib: n = 1, meloxicam: n = 1), minimal-dose prednisolone (1 dog treated for previously diagnosed hypoadrenocorticism), antimicrobials (amoxicillin/clavulanic acid: n = 3; enrofloxacin: n = 2; unknown: n = 1), and/or other medications including ephedrine (n = 1), osaterone (n = 1), and buprenorphine (n = 1).

*Control dogs* – Breeds included American Staffordshire terrier, Collie, Dachshund (each n = 1), and mixed breed (n = 2); none of the dogs were neutered. All five dogs had died or were euthanized for reasons unrelated to the urogenital tract, and prostatic tissue specimens were sampled during necropsy. Histopathology of prostatic tissues was unremarkable in all five dogs; thus, these dogs were determined to be “healthy controls”. Age-related conditions not known or reported to affect the urogenital tract (e.g., degenerative joint disease) were not considered as an exclusion criterion.

**Fig. 1:**
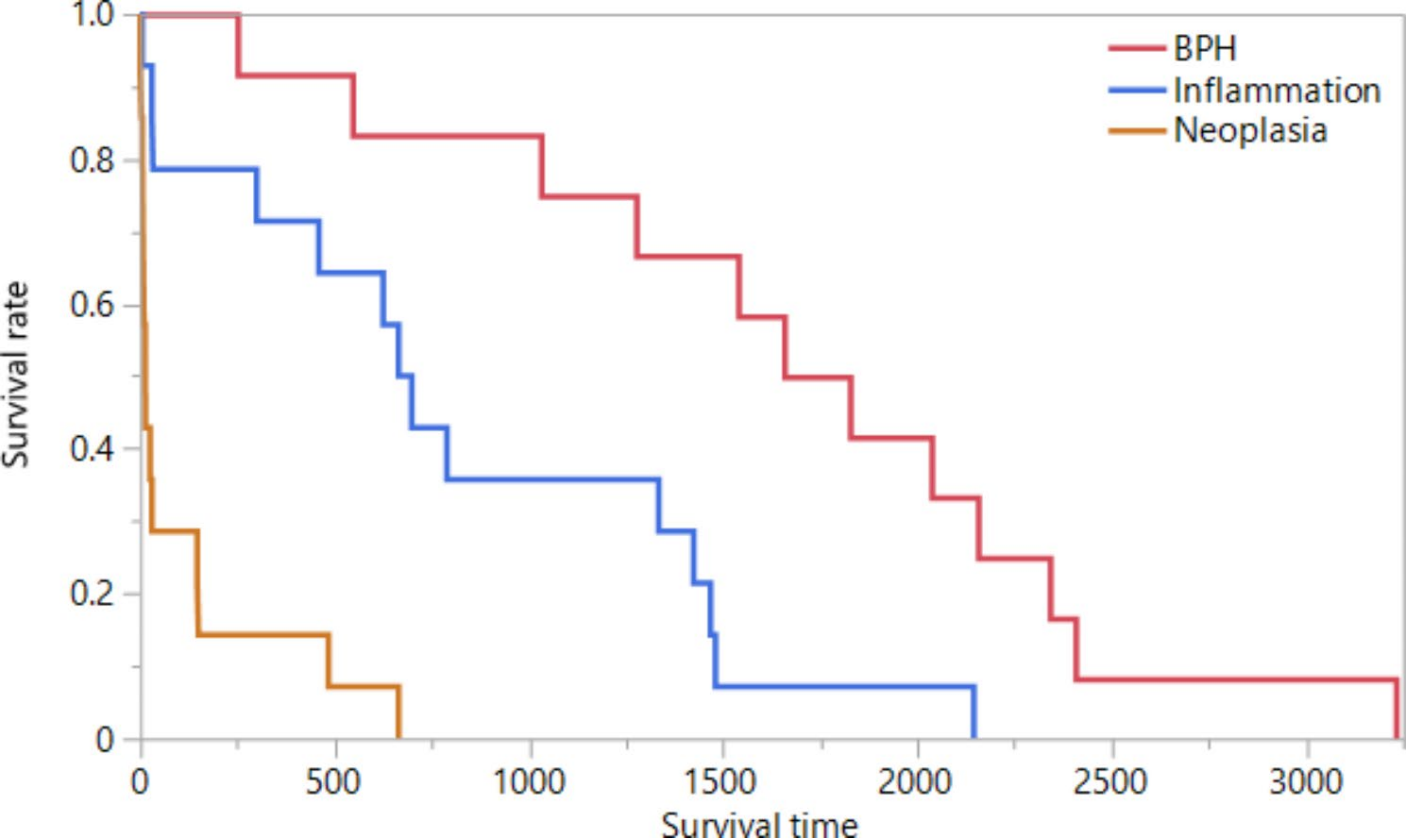
Kaplan-Meier survival plot for dogs with malignant versus benign prostatic conditions. Survival times (in days) in the dogs with prostatic neoplasia were significantly shorter (median: 14 days) than in dogs with severe prostatitis (median: 682 days; *P* = 0.0001) or BPH (median: 1,741 days; *P* < 0.0001) and were also significantly shorter in dogs with prostatitis compared to the BPH group (*P* = 0.0039)

Dogs with prostatic neoplasia and those diagnosed with marked prostatitis were significantly older (medians: 10.3 years and 10.0 years) than dogs in the control group (median: 5.0 years; *P* = 0.0160 and *P* = 0.0233, respectively). Dogs with BPH had significantly higher body weights than dogs with prostatic neoplasia (*P* = 0.0193), severe prostatitis (*P* = 0.0155), or controls (*P* = 0.0203). Dogs with prostatic neoplasia were significantly more likely to be neutered than dogs with BPH (*P* = 0.0079) or controls (*P* = 0.0108), but the lower rate of neutered dogs with severe prostatitis compared to prostatic neoplasia did not reach statistical significance (*P* = 0.0570). Survival times were significantly shorter in dogs with prostatic neoplasia compared to dogs with severe prostatitis (*P* = 0.0001) or BPH (*P* < 0.0001), with a significant difference also between dogs with prostatitis and those with BPH (*P* = 0.0039) (Table 1; Fig. 1); no observations were censored.

### S100/calgranulin immunohistochemistry

**Fig. 2:**
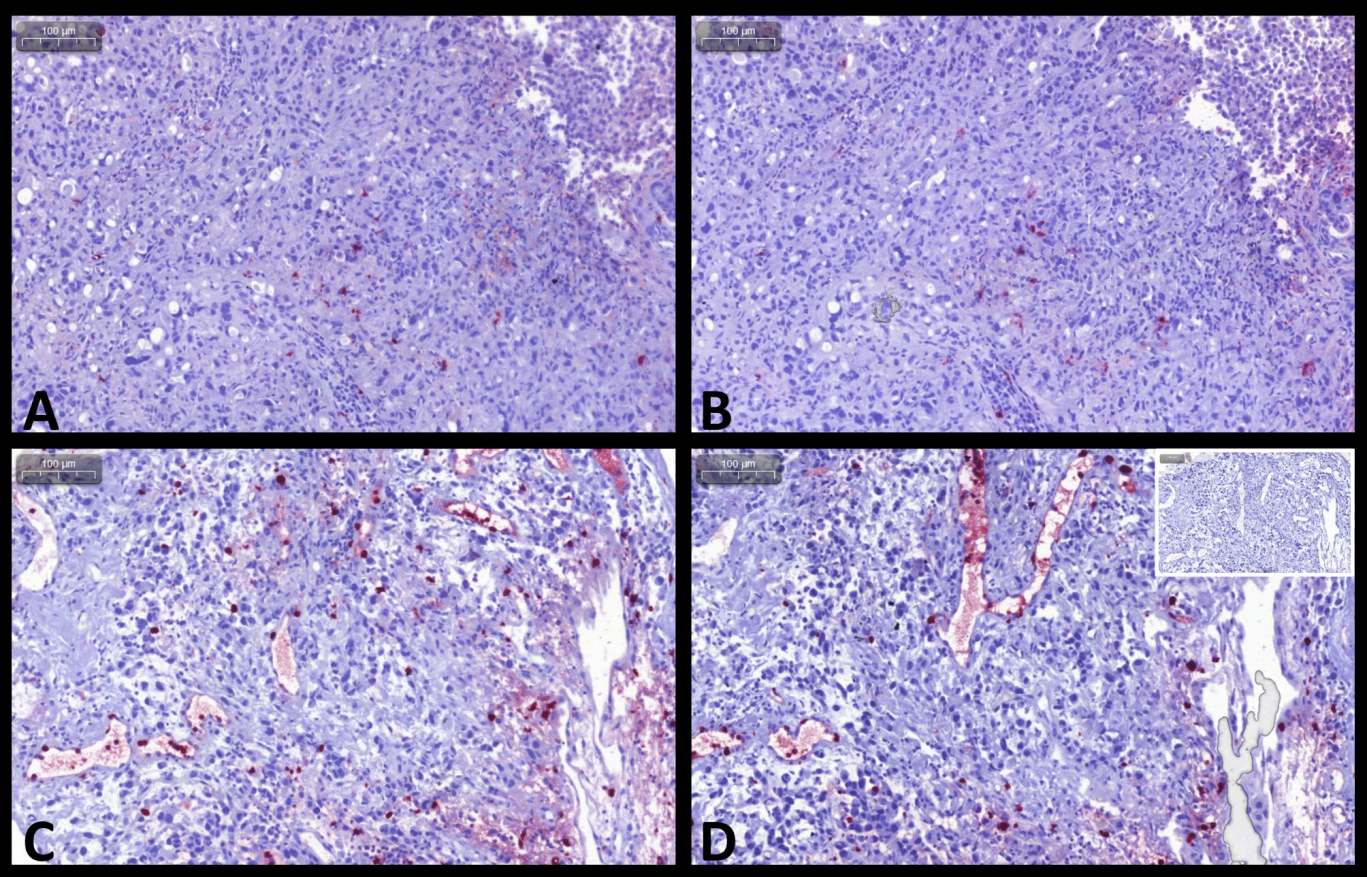
S100/calgranulin immunohistochemistry (IHC) of prostatic tissue biopsies in dogs with prostatic carcinoma. Upper panel: small numbers of infiltrating cells staining positive (Fast-red) for **(A)** S100A8/A9 or **(B)** S100A12 in a 10½-year old neutered Jack Russel terrier. Lower panel: moderate numbers of infiltrating cells staining positive for **(C)** S100A8/A9 or **(D)** S100A12 (insert at the bottom right image: negative staining control) in a 10½-year old male Maltese. Gray scale bars at the top left corners: 100 μm

**Fig. 3:**
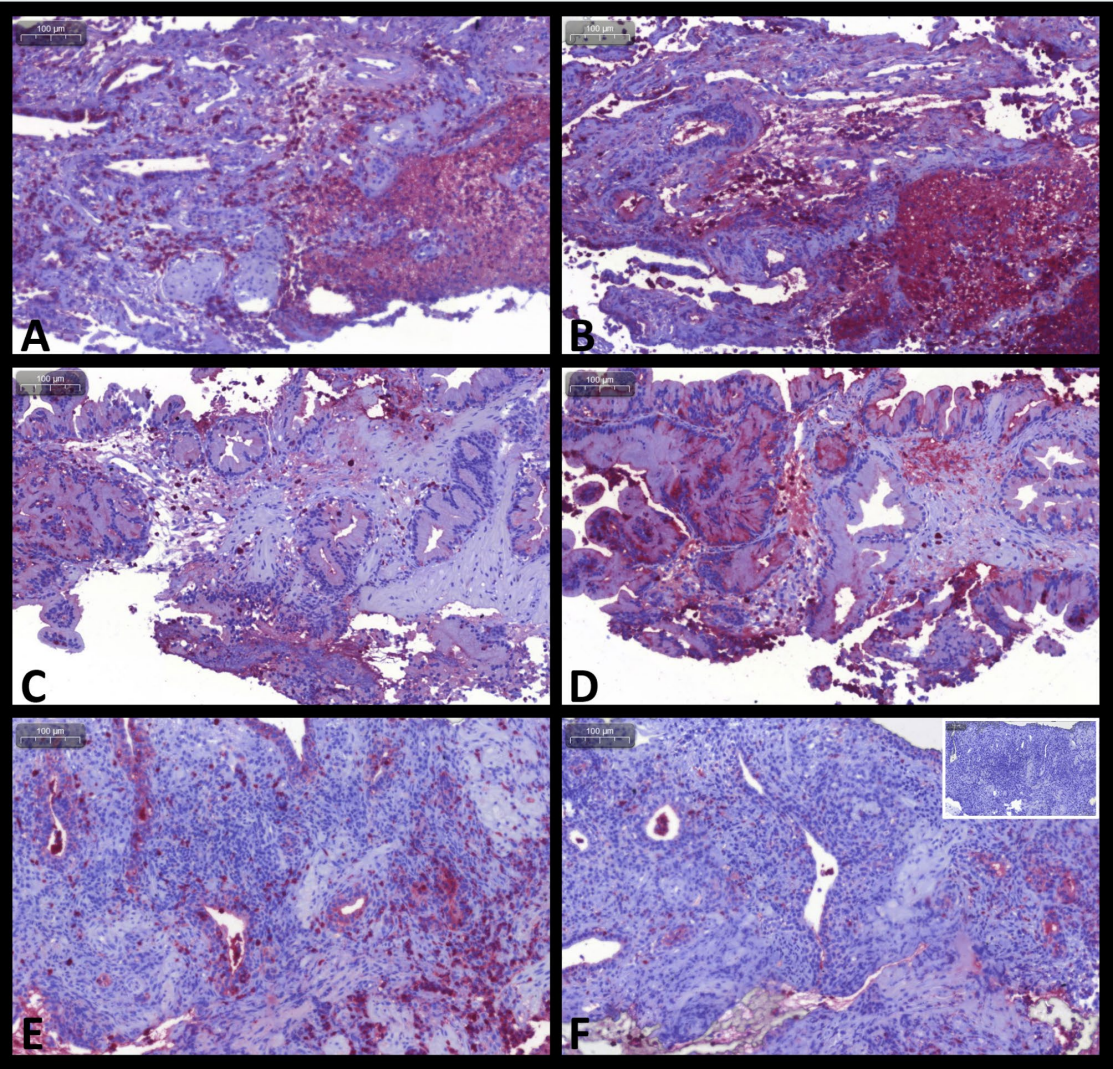
S100/calgranulin immunohistochemistry (IHC) of prostatic tissue biopsies in dogs with prostatitis. Upper and middle panel: large numbers and nest-like accumulations of infiltrating cells staining positive (Fast Red) for **(A, C)** S100A8/A9 or **(B, D)** S100A12 in a 9-year-old male German Shorthair pointer diagnosed with marked suppurative prostatitis. Lower panel: moderate numbers of infiltrating cells staining positive for **(E)** S100A8/A9 or **(F)** S100A12 (insert at the bottom right image: negative staining control) in a 6-year-old neutered male mixed breed diagnosed with severe chronic fibrosing prostatitis with cyst formation. Gray scale bars at the top left corners: 100 μm

**Fig. 4:**
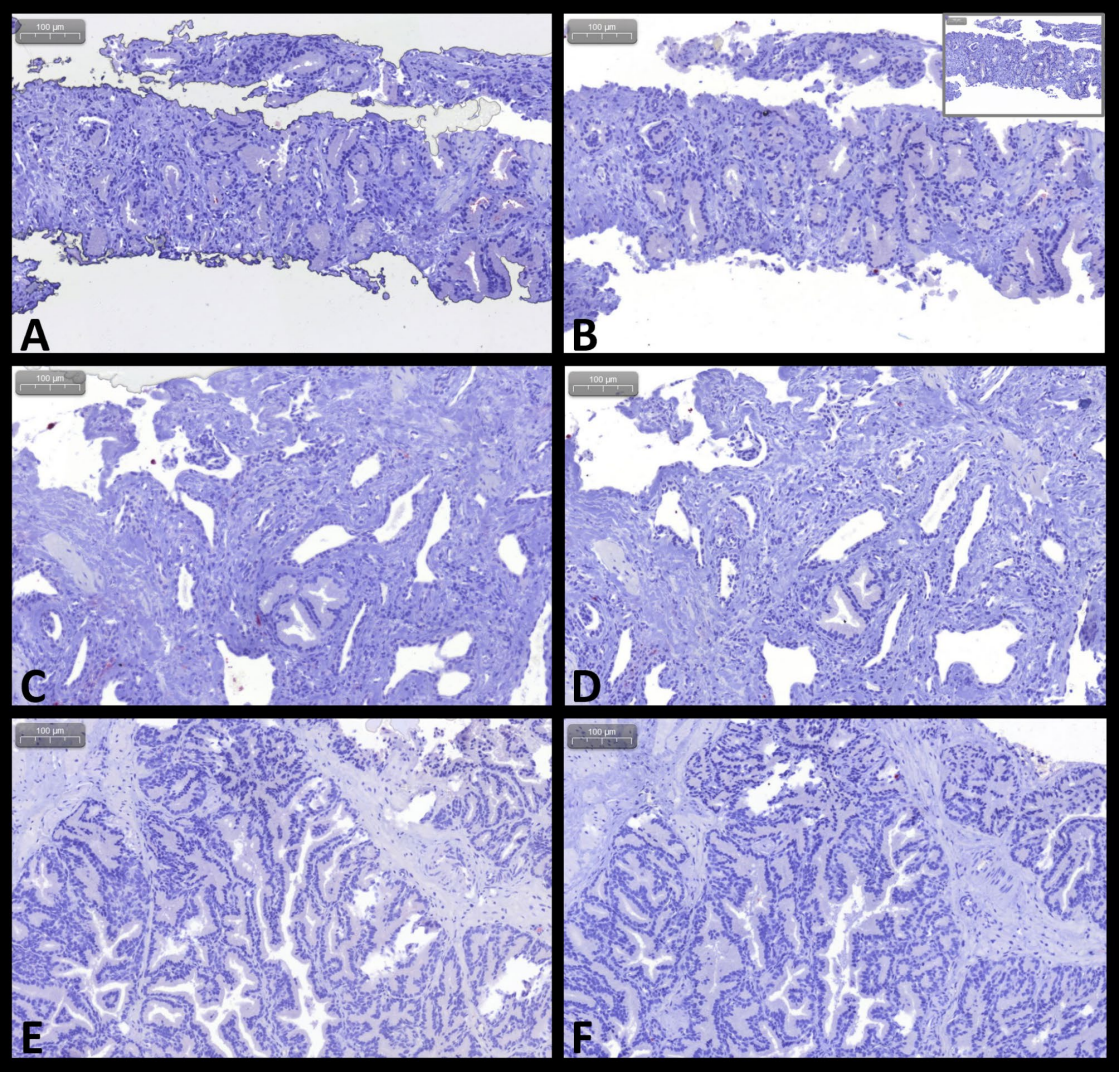
S100/calgranulin immunohistochemistry (IHC) of prostatic tissue biopsies in dogs with benign prostatic hyperplasia (BPH). Upper and middle panel: low numbers of infiltrating cells staining positive (Fast Red) for **(A, C)** S100A8/A9 or **(B, D)** S100A12 in a 7½-year-old male American bulldog (insert at the top right image: negative staining control). Lower panel: minimal to no staining for **(E)** S100A8/A9 and **(F)** S100A12 in a 2-year-old male Bernese Mountain dog. Gray scale bars at the top left corners: 100 μm

Overall comparisons of the numbers of cells staining positive for S100A8/A9, S100A12, and their ratio (Cal-ratio) are summarized in Table 2. A total of 32 sets of prostatic tissue biopsy samples were evaluated from nine dogs with prostatic neoplasia (Fig. 2; for one dog, only S100A12^+^ cell counts were evaluated due to insufficient tissue available for paired evaluation of the S100A8/A9^+^ cell counts), eight dogs with severe prostatitis (Fig. 3), ten dogs with BPH (Fig. 4), and five dogs comprising the control group. Based on the cell morphology, both S100A8/A9^+^ and S100A12^+^ cells were identified primarily as neutrophils and macrophages.

**Table 2:** S100A8/A9^+^ cell counts, S100A12^+^ cell counts, and the S100A8/A9^+^-to-S100A12^+^ cell counts ratio (Cal-ratio) in canine prostatic tissue biopsies (n = 32) with a diagnosis of prostatic adenocarcinoma or urothelial carcinoma (n = 9), marked prostatitis (n = 8), benign prostatic hyperplasia (n = 10), and healthy controls (n = 5)

Parameter	Prostatic neoplasia	Prostatitis	Benign prostatic hyperplasia	Control dogs	*P* value^†^
Total number of dogs	9	8	10	5	–
**S100A8/A9 expression**					
Tissue sections evaluated median [range]	6^#^ [5–7]	6 [6–7]	6 [6–7]	7 [7–7]	–
S100A8/A9^+^ cells/0.01 mm^2^ median [range]	**1** ^#,A,B^ [0–38]	**9** ^A^ [1–22]	**1** ^B^ [0–2]	**0** ^C^ [0]	**0.0006**
**S100A12 expression**					
Tissue sections evaluated median [range]	6 [5–6]	6 [6–7]	6 [6–7]	7 [6–7]	–
S100A12^+^ cells/0.01 mm^2^ median [range]	**1** ^A,B^ [0–40]	**9** ^C^ [0–17]	**1** ^A^ [0–4]	**0** ^B^ [0]	**0.0041**
**S100A8/A9-to-S100A12 ratio**					
Cal-ratio median [range]	1.0 [0.5–2.9]	1.3 [0.5–2.0]	0.9 [0.4–1.8]	1.0 [1.0]	0.2419

^†^bold font indicates significance at *P* < 0.05, and values with a different superscript letter differ significantly at *P* < 0.05; ^#^only available from 8 dogs

Mean S100A8/A9^+^ cell counts were higher (median: 9 cells/0.01 mm^2^) in dogs with marked prostatitis compared to the other three groups of dogs (medians: 0–1 cell/ 0.01 mm^2^; Table 2). However, statistical significance was reached only for the difference of prostatitis with BPH (*P* = 0.0013) and controls (*P* = 0.0033) but not when compared to neoplastic disease (*P* = 0.0659). S100A8/A9^+^ cell counts were also significantly higher in neoplastic tissue compared to healthy control tissues (*P* = 0.0210) but not in comparison to the BPH group (*P* = 0.2612). Mean numbers of tissue S100A12^+^ cells were significantly higher in dogs with severe prostatitis (median: 9 cells/0.01 mm^2^) compared to dogs with prostate neoplasia (median: 1 cell/0.01 mm^2^; *P* = 0.0458), BPH (median: 1 cell/0.01 mm^2^; *P* = 0.0163), or controls (median: 0 cells/0.01 mm^2^; *P* = 0.0089). No significant differences in tissue S100A12^+^ cell counts were detected between dogs with prostatic neoplasia and dogs with BPH (*P* = 0.6499) or controls (*P* = 0.0622; Table 2). The ratio between the number of S100A8/A9^+^ cells and S100A12^+^ cell counts (Cal-ratio) was also highest in the group of dogs with marked prostatitis, but the Cal-ratio did not differ significantly among the four groups of dogs (all *P* > 0.05). However, significantly higher Cal-ratios were detected in dogs with marked prostatitis that died or were euthanized (median: 1.56, n = 6) than dogs that were still alive at the end of the study (median: 0.68, n = 2; *P* = 0.0455). A possible association of metastatic disease with S100A8/A9^+^ cell counts, S100A12^+^ cell counts, or Cal-ratios could not be evaluated due to most dogs (8/9, 89%) having suspected or confirmed metastatic disease at the time when prostatic neoplasia was diagnosed.

Evaluating all three disease groups combined, dogs with a positive urine culture had significantly higher S100A8/A9^+^ cell counts (median: 6 cells/ 0.01 mm^2^, n = 8) and S100A12^+^ cell counts (median: 5 cells/0.01 mm^2^, n = 8) than dogs with a negative urine culture (median: 0 cells/0.01 mm^2^, n = 11; *P* = 0.0158 and median: 0 cells/0.01 mm^2^, n = 12; *P* = 0.0065). Similarly, positive prostatic tissue cultures were linked to significantly higher S100A8/A9^+^ cell counts (median: 11 cells/0.01 mm^2^, n = 3) and S100A12^+^ cell counts (median: 11 cells/0.01 mm^2^, n = 3) than negative prostatic culture results (median: 0 cells/0.01 mm^2^, n = 12; *P* = 0.0098 and median: 0 cells/0.01 mm^2^, n = 12; *P* = 0.0152).

### Neutrophil-to-lymphocyte ratio (NLR)

Neutrophil counts were highest, and lymphocyte counts lowest, in dogs with marked prostatitis, but neither the individual cell counts nor the NLR differed significantly among the three disease groups of dogs (all *P* > 0.05; Table 3). The NLR was strongly correlated with the number of S100A8/A9^+^ cells in prostatic tissue biopsies from dogs with prostatic neoplasia (ρ = 0.81, *P* = 0.0499) and the numbers of prostatic tissue S100A12^+^ cells in dogs with BPH (ρ = 0.93, *P* = 0.0025), but not with any S100/calgranulin-positive cell counts in dogs with marked prostatitis (all *P* > 0.05). Positive cultures of urine or prostatic tissue biopsies were not associated with blood neutrophil counts, lymphocyte counts, or the NLR (all *P* > 0.05), but this could not be analyzed separately for the different study groups due to the small sample size.

**Table 3:** Neutrophil and lymphocyte counts and the neutrophil-to-lymphocyte ratio (NLR) in dogs with neoplastic or non-neoplastic prostatic diseases (n = 34)

Parameter	Prostatic neoplasia	Prostatitis	Benign prostatic hyperplasia	*P*-value
Total number of dogs	12	13	9	–
Neutrophil count, in 10^9^/L median [range]	6.90 [3.83–37.88]	7.71 [0.26–23.40]	8.77 [5.22–16.66]	0.6784
Lymphocyte count, in 10^9^/L median [range]	1.62 [0.96–2.99]	1.30 [0.77–3.27]	1.29 [0.91–3.08]	0.5705
NLR median [range]	4.8 [1.9–23.0]	6.5 [0.2–13.3]	5.5 [2.9–15.2]	0.9085
NLR increase^#^, n (%)	6 (50%)	10 (77%)	6 (67%)	0.3659

^#^defined as an NLR > 4.1 [40]

## Discussion

The present study followed previous investigations by our group [46] and further evaluated the source and cellular distribution of S100/calgranulin secretion into urine and a possible association with systemic leukocyte responses reflected by NLR changes. To the authors’ knowledge, this is the first study evaluating S100A8/A9 and S100A12 in canine prostate tissues and the blood NLR in common prostatic diseases in dogs. In the previous study, urinary concentrations of S100A8/A9 and S100A12 were significantly increased in dogs with UC, PCA, or UTI compared to healthy control dogs. Furthermore, the urinary Cal-ratio was significantly lower in dogs with UTI than in the other two disease groups of dogs, suggesting its potential as a screening test [25, 46]. In line with the present study, our previous evaluation of tissue samples from dogs revealed no significant difference in the numbers of S100/calgranulin-expressing cells between inflammatory and neoplastic lower urinary tract conditions. However, tissue S100A12^+^ cell counts were significantly higher in both disease groups compared to healthy controls, resulting in significantly lower Cal-ratios in dogs with UC or non-neoplastic urinary tract diseases (NNUTD) compared to normal lower urinary tract tissues [42]. The present study identified significantly higher S100A8/A9^+^ cell counts in tissue samples from dogs with prostatitis compared to BPH and healthy controls, and dogs with PCA had significantly higher numbers of S100A8/A9^+^ cells compared to healthy controls but not in comparison to the other disease groups. In contrast, BPH produced significantly higher S100A12^+^ cell counts than healthy controls but significantly lower numbers than in dogs with prostatitis. This could be explained by the presence of minimal to mild non-suppurative prostatic inflammation in some BPH cases. Prostatitis was associated with significantly higher tissue S100A12^+^ cell counts compared to all other groups, with no significant difference between dogs with PCA, BPH, or controls. Finding significantly more cells that stained positive for S100A8/A9 and S100A12 in dogs with an inflammatory condition than in control tissues resembles, to some extent, the results of the previous study on urine samples [46]. The findings are consistent with higher urinary S100/calgranulin concentrations in dogs with UTI [25] and higher tissue S100/calgranulin-positive cell counts in NNUTD cases than in healthy controls [42]. Similar to our previous investigation, increased numbers of tissue S100/calgranulin-expressing cells were also detected in dogs with confirmed UTI.

In contrast to the previous study on canine urine samples [46], tissue S100/calgranulin-positive cell counts were not significantly higher in dogs with PCA than in those with prostatitis. This agrees with tissue S100A8/A9^+^ cell counts not differing between cases of UC, NNUTD, and controls, and urinary S100A12 concentrations and tissue S100A12^+^ cell counts being increased compared to healthy controls [25, 42]. This again suggests that the number of S100/calgranulin-expressing cells does not reflect the amount of S100/calgranulins released from affected tissues and into the urine. An alternative explanation for the discrepancy between these studies might be an effect of pre-treatment (particularly NSAIDs), which differed between the study populations with fewer treatment-naïve dogs evaluated for tissue S100/calgranulin expression. Similar to cyclooxygenase (COX)-2 being overexpressed in canine UC cells [47], most canine prostatic carcinomas also express COX-2, whereas this enzyme is not expressed in normal prostatic tissue [22, 48, 49], and COX-2 inhibitors (NSAIDs) are used for PCA treatment with acceptable side effects [22]. Further research is necessary to clarify the possible role of COX-inhibitor treatment on S100/calgranulin expression in canine PCA. Furthermore, the study on urinary S100/calgranulin concentrations evaluated cases of UC and PCA combined in one group [25], and tissue S100A12^+^ cell counts were higher in the UC group than in NNUTD cases [42], which might have contributed to higher urinary S100A12 concentrations in dogs with urogenital neoplasia irrespective of the contribution of prostatic cancer cases.

The results for the Cal-ratio differed between all three studies in dogs. There was no significant difference in the Cal-ratio among the four groups of dogs in this study, whereas tissue Cal-ratios were significantly lower in UC and NNUTD compared to healthy controls [42], and the urinary Cal-ratios were even significantly lower with UTI compared to neoplastic conditions and controls [25]. A likely explanation is that the amounts of S100/calgranulin proteins produced and released by these cells vary and do not resemble the number of infiltrating S100/calgranulin-positive cells. However, further research is needed to confirm this assumption. Furthermore, the urinary results might have been affected by the combined analysis of urogenital neoplasia cases [25], and distinct molecular pathways might be involved in the tumorigenesis of these neoplastic conditions and/or in the different tumor stages [50]. S100/calgranulin expression might also vary among the different organ systems or segments within the same organ system, accounting for potential differences in prostatic vs. urinary bladder/urethral UC. Prostatic carcinoma and UC cases were included and evaluated together in this study, reflecting the clinical scenario of both being challenging to distinguish and the focus on improving the non-invasive differentiation of neoplastic from benign lesions in a grossly (e.g., ultrasonographically) altered prostate. Another possible explanation for the discrepancies between different studies could be the inherent difficulty in documenting high-grade prostatic intraepithelial neoplasia (PIN) as a potential precursor to canine PCA [7, 51, 52]. With a total of four core biopsies routinely obtained (1 biopsy from each quadrant of the prostate), there remains a risk that high-grade PIN (or even PCA) could be missed in the remaining prostatic tissue, which in turn might affect S100/calgranulin synthesis and release.

Tissue Cal-ratios were significantly higher in non-survivors compared to survivors in the prostatitis group. This leads us to suspect that S100/calgranulin (particularly S100A8/A9) expression, may have prognostic value in canine prostatic inflammation as has been shown for prostatic cancer in men [53] and could result from exacerbated inflammation through pathways down-stream from S100A8/A9 [54]. Further evaluation of this hypothesis and the potential prognostic value of the S100/calgranulins and/or their potential role in targeted therapeutics in canine prostatitis are thus warranted.

Dogs in the PCA and prostatitis groups were significantly older than those dogs in the healthy control group, and dogs with prostatitis were significantly older than the BPH group. This agrees with the literature showing that PCA is diagnosed in older dogs, whereas BPH is detected in a slightly younger population with > 80% of intact male dogs ≥ 5 years of age having BPH [10], and that there is no age predisposition for prostatitis. Lack of an age difference between PCA and prostatitis cases is important, given the primary interest in distinguishing malignant from benign prostatic conditions in elderly dogs. To the authors’ knowledge, there are no studies evaluating age-dependent prostatic S100/calgranulin expression, but the possibility of an effect of the different age distribution between BPH and prostatitis cases on tissue S100/calgranulin expression cannot be excluded.

Dogs with PCA were more likely to be neutered than controls, which is consistent with neutered dogs having a higher risk of developing PCA than intact dogs [8, 11, 12]. An interesting finding is that intact dogs did not show a higher risk of developing prostatitis. However, given the retrospective nature of the study, it remains unknown at which age the dogs were neutered, and it is possible that individual dogs were neutered because of a prostatic condition not long before being referred for biopsy sampling rather than developing prostatitis or BPH absent any androgen effects (i.e., under early-neuter status). In addition to age affecting prostatic S100/calgranulin expression as shown in men [55], an effect of the reproductive status cannot be excluded in our study population. A recent study evaluating possible associations of several patient characteristics and lifestyle factors with S100/calgranulin concentrations in feces from healthy dogs established a link with the reproductive status in female dogs but no correlation with sex or reproductive status in male dogs [56]. However, the possibility of sex and reproductive status affecting urogenital S100/calgranulin expression or urine S100/calgranulin concentrations has not been investigated.

Most dogs in this study had confirmed or suspected metastatic disease, which agrees with the literature reporting an > 40% rate of metastasis at the time of diagnosis (> 80% at the time of death), and common metastatic sites include the spleen, liver, and lymph nodes [4, 9, 13]. Testicular neoplasia was suspected in one dog, presenting either a concurrent primary testicular neoplasm or (less likely) an unusual metastatic process. PCA with a concurrent Sertoli cell tumor has been described [57].

Blood NLR could not differentiate among the disease groups in this study and does not appear to be a clinically useful marker to distinguish neoplastic from non-neoplastic prostatic diseases. This result contrasts with the findings in dogs with lower urinary tract disease in our previous study [42], but blood neutrophil counts might have been generally lower in the inflammatory group in this study as some dogs were clinically severely affected (e.g., one dog underwent laparotomy due to septic peritonitis and sepsis). However, blood NLRs strongly correlated with S100A8/A9^+^ cell counts in neoplastic tissues and S100A12^+^ cell counts in tissues from dogs with BPH. This is consistent with a systemic response induced by inflammatory processes (tumor inflammation, chronic inflammation with or without infection) and with systemic inflammation that extends from the inflammatory tumor microenvironment which plays an important (e.g., prognostic) role [58].

S100A8/A9 is primarily expressed in neutrophils and activated (tissue-infiltrating) macrophages but can also be induced in epithelial cells (e.g., with infection or neoplastic transformation) and can have pleiotropic roles [29, 59, 60]. The production of S100A12 appears to be more specific to innate inflammatory cells [28, 61]. Thus, it can be presumed that the S100/calgranulins are linked to the inflammatory environment, potentially affecting the inflammasome [32, 62–64] that can modulate tumor development, progression, and metastasis [65, 66]. Further investigation of the S100/calgranulin pathways in PCA is warranted.

We acknowledge the limitation of the present study to include retrospective cases, small numbers of dogs in each group, and not exclusively treatment-naïve canine patients. Also, samples were not all obtained using a standard approach to tissue biopsy collection, with tissues obtained via USGTC, surgically, or during a necropsy, but the effect on the results of this study is regarded as negligible. Further, given our aim to include histologically normal prostatic tissues as an additional control group to BPH cases and the strict inclusion criteria applied for this group, significantly younger animals were included in the healthy control group compared to the remaining groups of dogs. Lastly, several pathologists were involved in the routine histologic evaluation of the tissues, potentially causing inter-observer variation. However, a single pathologist not involved in the routine diagnostic evaluation (CG) re-examined all tissue specimens at the time of IHC analysis without differing interpretations or diagnoses.

## Conclusions

Tissue expression of the S100/calgranulins varied among malignant and benign conditions of the prostate in dogs, but did not differ significantly between PCA and prostatitis, with similar patterns as in canine lower urinary tract diseases. However, there are discrepancies when concentrations in urine samples of dogs diagnosed with these conditions are compared. Presumably, the S100/calgranulins have varying roles in these diseases and might be linked to the inflammatory microenvironment, potentially affecting the inflammasome in dogs with prostatic neoplasia. Thus, S100/calgranulin pathways and their possible role in modulating tumor development, progression, and metastasis warrant further study. The blood NLR does not appear to serve as a useful marker to distinguish malignant from benign diseases of the prostate. However, similarly to tissue S100A8/A9^+^ cell counts, the NLR might be of value for further stratification of the population of dogs with prostatic cancer. The possibility of an effect of pre-treatment and individual patient characteristics on tissue S100/calgranulin expression and their release into the urine also requires further investigation.

## Methods

### Ethics approval

All methods were carried out following the relevant guidelines and EU regulations and are reported in accordance with the ARRIVE guidelines (www.arriveguidelines.org). The protocol for the collection of tissue samples from dogs with urogenital tract disease and healthy dogs (institutional animal use protocol) was independently reviewed and approved by the Regional Veterinary Council (Animal Experimentation/Ethics Committee, acc. § 15 EU/German Animal Welfare Law) of the Free State of Saxony, Leipzig/Chemnitz, SN, Germany (TVA# 23/18, approved 11-28-2018), and written consent was obtained from the owners of all dogs that were prospectively enrolled into the study. For surplus specimens and medical records data of dogs that were retrospectively included in the study, written owner consent for the use of surplus samples and patient data was obtained on the small animal clinic’s standard patient admission form at the time of the first presentation of the dog for routine diagnostic evaluation.

### Sampling population

Canine patients (n = 45) for the case-control study were recruited at the Department for Small Animals and the Institute of Veterinary Pathology, University of Leipzig College of Veterinary Medicine. Inclusion criteria were histopathology-confirmed PCA (n = 14), marked prostatitis (n = 14), BPH (n = 12), or a histologically normal prostate (controls; n = 5), and sufficient archived tissue material to perform IHC. Of those, ten dogs were prospectively enrolled, and archived tissue biopsy specimens and/or patient medical records data were retrospectively included from the remaining 35 animals; all control tissues were obtained during a necropsy after the death due to an unrelated cause (Table 1). Pre-treatment was not an exclusion criterion except for chemotherapy. Complete patient information was extracted from the electronic medical records (for retrospectively enrolled cases) and/or a standard study questionnaire completed by the owner and/or the attending veterinarian at patient enrolment (for all prospectively enrolled cases). The referring veterinarian, the dog owners, or both were consulted via telephone to obtain follow-up information using a standardized study questionnaire.

### Tissue sample analyses

Prostatic tissue specimens from dogs with PCA (n = 9), marked prostatitis (n = 8), BPH with or without minimal to mild prostatitis (n = 10), and controls (n = 5) were used for the tissue S100/calgranulin expression study (Table 2). These tissue samples were obtained via minimally invasive TruCut biopsy (n = 27) or surgical wedge biopsy with a laparotomy approach to the prostate (n = 13) or during autopsy (n = 5). Of the 27 patients with prostatic disease, five dogs were treatment-naïve (19%), whereas 22 dogs (81%) had received an NSAID (n = 15), antimicrobial (n = 18), and/or other treatment (n = 7) prior to biopsy sampling.

After sampling, the tissue specimens were fixed in neutral-buffered formaldehyde (4%), paraffin-embedded, cut into 3-µm slices, and placed on microscopy slides. Routine histopathology of the urinary tract tissues was performed on hematoxylin/eosin-stained slides by one of six board-certified and/or nationally accredited veterinary anatomic pathologists. After obtaining a histologic diagnosis, 3-µm tissue re-cuts were prepared for IHC.

### Immunohistochemistry

The specimens (three slides of tissue samples from each dog and tissue) were deparaffinized in xylene and rehydrated in an ethanol series, followed by S100/calgranulin-IHC as previously described [42]. Briefly, after washing the slides in phosphate-buffered saline (PBS) with Tween 20 (0.025% v/v; PBST), heat-induced antibody retrieval was performed in 0.01 M citrate buffer (pH 6.0) at 95 °C for 45 min followed by cooling at room temperature (approximately 20 °C) for 20 min. Slides were washed twice in PBST and were incubated for 25 min with 4% bovine serum albumin (BSA) in PBS to prevent non-specific binding. Samples were then incubated overnight at 4 °C with the primary antibody (rabbit polyclonal anti-canine S100A8/A9 at 0.2 µg/mL or rabbit polyclonal anti-canine S100A12 at 0.25 µg/mL) [67, 68]; normal rabbit serum (0.2 µg/mL) served as a negative staining control. Slides were washed twice in PBST and incubated with the secondary antibody^1^ (1.0 µg/mL) for 60 min at room temperature. After two PBST washes, the slides were incubated with Fast Red^2^ for 30 min, followed by sequential washing in PBST and ddH_2_O after optimal color development. Mayer’s hematoxylin was used to counterstain the nuclei, and the slides were mounted^3^. After cursory assessment by light microscopy, the slides were digitized using the Pannoramic Scan II^4^ with a 20× objective lens. The digital images were then examined by a board-certified veterinary pathologist (CG) in a blinded fashion using the CaseViewer digital microscopy application^5^.

After re-evaluating the histopathologic diagnosis, distribution of positive staining, and the absence of staining in the corresponding negative control, 5–7 regions of 0.01 mm^2^ were randomly selected based on optimal tissue integrity and orientation for evaluation. In these regions, all S100A8/A9-positive (S100A8/A9^+^) and S100A12-positive (S100A12^+^) staining cells were identified and were counted if yielding a cytoplasmic and/or membranous IHC signal. For data analysis, the average numbers of S100A8/A9^+^ cells and S100A12^+^ cells were calculated for all regions evaluated from the same dog, and the S100A8/A9-to-S100A12 ratio (Cal-ratio) was determined as [(average number of S100A8/A9^+^ cells) / (average number of S100A12^+^ cells)].

### Blood leukocyte analysis

Complete blood cell counts (CBC) were performed using an automated blood cell analyzer. Neutrophil counts and lymphocyte counts were measured in ×10^9^ cells/L (reference intervals: 3.0–11.6 × 10^9^/L and 1.0–5.1 × 10^9^/L), and the neutrophil-to-lymphocyte ratio (NLR; reference interval: 1.0–4.1) was calculated as [(neutrophil count) / (lymphocyte count)] [40].

### Data analyses

A commercially available statistical software package^6^ was used for all statistical analyses. Data were tested for the assumption of normality using a Shapiro-Wilk test. Summary statistics are reported as medians and ranges (continuous data) or counts and percentages (categorical data). Continuous data (age, body weight, S100A8/A9^+^ and S100A12^+^ cell counts, Cal-ratio, neutrophil and lymphocyte counts, NLR) were compared among or between the three or four different groups of dogs using non-parametric multiple- (Kruskal-Wallis test) or two-group (Mann-Whitney *U* test) comparisons. Categorical data (neuter status) were compared using a likelihood ratio or Fisher’s exact test. A non-parametric Spearman correlation coefficient (ρ) served to test the relationship between continuous variables. Survival curves were plotted using the Kaplan-Meier method, and the log-rank test was used for survival analysis between groups. Statistical significance was set at *P* < 0.05.

## Data Availability

The datasets used and/or analyzed during the current study are available from the corresponding author upon reasonable request.

## Footnotes

1 Goat anti-rabbit alkaline phosphatase-labeled immunoglobulin, Dianova, Hamburg, Germany.

2 SigmaFast™ Fast Red TR/Naphthol AS, Merck, Darmstadt, Germany.

3 Fluoromount-G™ Mounting Medium, Thermo Fisher, Dreieich, Germany.

4 Pannoramic Scan II, 3D-Histech Ltd., Budapest, Hungary.

5 CaseViewer digital microscopy application, Slides.3D-Histech Ltd., Budapest, Hungary.

6 JMP v13.0, SAS Institute Inc., Cary, NC, USA.

## Abbreviations

BSA: Bovine serum albumin
BPH: Benign prostatic hyperplasia
Cal-ratio: S100A8/A9-to-S100A12 ratio
COX-2: Cyclooxygenase-2
NLR: Neutrophil-to-lymphocyte ratio
NNUTD: Non-neoplastic urinary tract disease
PBS: Phosphate-buffered saline
PBST: PBS with Tween
PCA: Prostatic carcinoma
ROC: Receiver operating characteristic curve
S100A8/A9: S100A8/A9 (calprotectin) protein complex
S100A12: S100A12 protein
UTI: Urinary tract infection
UC: Urothelial carcinoma
IHC: Immunohistochemistry
NSAID: Non-steroidal anti-inflammatory drug
